# Survivin, a molecular target for therapeutic interventions in squamous cell carcinoma

**DOI:** 10.1186/s11658-017-0038-0

**Published:** 2017-04-05

**Authors:** Zakir Khan, Abdul Arif Khan, Hariom Yadav, Godavarthi B. K. S. Prasad, Prakash Singh Bisen

**Affiliations:** 1 School of Studies in Biotechnology, Jiwaji University, Gwalior, 474001 MP India; 2 Department of Biomedical Sciences, Department of Pathology, Cedars-Sinai Medical Center, Los Angeles, CA 90048 USA; 3 Department of Pharmaceutics, College of Pharmacy, King Saud University, Riyadh, Saudi Arabia; 4 National Institute of Diabetes and Digestive and Kidney Diseases, National Institutes of Health, Bethesda, MD 20892 USA

**Keywords:** Squamous cell carcinoma (SCC), Survivin, Apoptosis, Cancer immunotherapy, YM155, Hsp90 inhibitors, CDK inhibitors

## Abstract

Squamous cell carcinoma (SCC) is the most common cancer worldwide. The treatment of locally advanced disease generally requires various combinations of radiotherapy, surgery, and systemic therapy. Despite aggressive multimodal treatment, most of the patients relapse. Identification of molecules that sustain cancer cell growth and survival has made molecular targeting a feasible therapeutic strategy. Survivin is a member of the Inhibitor of Apoptosis Protein (IAP) family, which is overexpressed in most of the malignancies including SCC and totally absent in most of the normal tissues. This feature makes survivin an ideal target for cancer therapy. It orchestrates several important mechanisms to support cancer cell survival including inhibition of apoptosis and regulation of cell division. Overexpression of survivin in tumors is also associated with poor prognosis, aggressive tumor behavior, resistance to therapy, and high tumor recurrence. Various strategies have been developed to target survivin expression in cancer cells, and their effects on apoptosis induction and tumor growth attenuation have been demonstrated. In this review, we discuss recent advances in therapeutic potential of survivin in cancer treatment.

## Background

Squamous cell carcinoma represents one of the most common cancers worldwide. It is a malignancy that arises from uncontrolled growth of epithelial cells [1], and normally occur in the organs that covered with squamous epithelium [2]. Major types of SCC include head and neck cancer (HNSCC), esophageal cancer (ESCC), non-melanoma skin cancer, and non-small cell lung cancer (NSCLC) [2]. SCC is associated with greater mortality and morbidity due to its highly invasive nature that often invades neighboring tissues, and can metastasize distant organs [3, 4]. In advanced stages, SCC treatment often requires complete excision of tumor using specialized surgical techniques [5]. Unfortunately, the survival rate of SCC patients has not improved significantly over the last couple of decades, even after substantial advances in cancer treatment strategies [6].

In recent times, molecular targets that are involved in the regulation of cell death or viability pathways in cancer cell took a center stage in molecular cancer therapy research [7]. Other than bcl-2 family proteins [8], a second gene family called IAP has been identified, which regulates various important aspects of cell survival [9]. IAP family proteins are evolutionarily highly conserved, which exist from viruses to mammalian cells [10]. These proteins target downstream steps of apoptosis by interfering in the activation of pro- and effector caspases [11, 12]. The present article reviews the therapeutic potential of an IAP family protein survivin in cancer with special reference to SCC.

## Survivin

Survivin is a unique member of the IAP family that is expressed in most human tumors, but is barely detected in normal adult tissues [13, 14]. It is included in among the top five tumor-specific genes [15]. Overexpression of survivin in tumors is generally associated with poor prognosis and drug resistance [16, 17]. Nuclear expression of survivin has been established as a good prognostic marker in several cancers [18, 19]. Down-regulation of survivin induces cancer cell apoptosis, and suppresses tumor growth.

Survivin is the smallest member of the mammalian IAP family containing only a single N-terminal baculovirus IAP repeat (BIR) domain combined with long C-terminal α -helix coiled region [20]. In solution it is present in dimeric form. BIR domain plays a critical role in anti-apoptotic functions of survivin, whereas the coiled domain helps survivin in interacting with tubulin structures, and probably is involved in the regulation of cell division [21, 22]. A typical BIR domain consists of approximately 70 amino acids. The sequence and structure of BIR domain are evolutionarily highly conserved. For example, TIAP of murine and deterin of *Drosophila melanogaster* fruit flies show similarity with survivin [23]. Likewise, the genomes of *Xenopus laevis*, African clawed frog, *Xenopus tropicalis,* Western clawed frog, *Danio rerio*, zebra fish, *fugu,* puffer fish, and Oncorhynchus mykiss rainbow trout contain two genes Su1 and Su2 that are similar to survivin. The human survivin is a 16.5 kDa protein, which is encoded by BRIC5 gene and spans 14.7 kb at the telomeric position of chromosome 17 [24, 25].

Survivin gene also shows alternative transcriptional splicing that forms several of its isoforms [26]. These splice variants are formed with deletion and insertion of some of the coding and noncoding sequences, which are not much different in length from survivin [27]. Survivin-2B transcript is formed due to retention of a part of intron 2, while that of survivin-ΔEx-3 is formed by deletion of a part of exon 3. An insertion of additional exon 3B was found in survivin-3B transcript that leads to a frameshift and premature termination of the protein [22, 28]. The sequence alterations in different isoforms cause structural changes in the corresponding protein, consequently changing their ability to inhibit apoptosis. In case of survivin-2B, insertion of exon 2B at the site of essential BIR sequence reduces its anti-apoptotic function, whereas survivin-ΔEx-3 still retains the anti-apoptotic activity despite having slight alteration in BIR domain due to removal of exon 3. Different subcellular localizations are also observed within survivin isoforms. Survivin-ΔEx-3 is predominately nuclear, whereas survivin and survivin-2B are primarily present in cytoplasm [29]. Thus, the formation of different isoforms of survivin and their different subcellular localizations provide diversity to its functions.

### Functions of survivin

In cancer cells, survivin has two major functions; 1) regulation of mitosis by forming chromosomal passenger complex (CPC) with other proteins, and 2) inhibition of apoptosis [30, 31]. As shown by embryonic lethality in mice with survivin locus disruption that it plays a critical in overall normal embryonic development [32]. In adults, survivin is absent in most of the terminally differentiated tissues as opposed to it high re-expression in malignant cells.

### Role of survivin in cell division

**Fig. 1:**
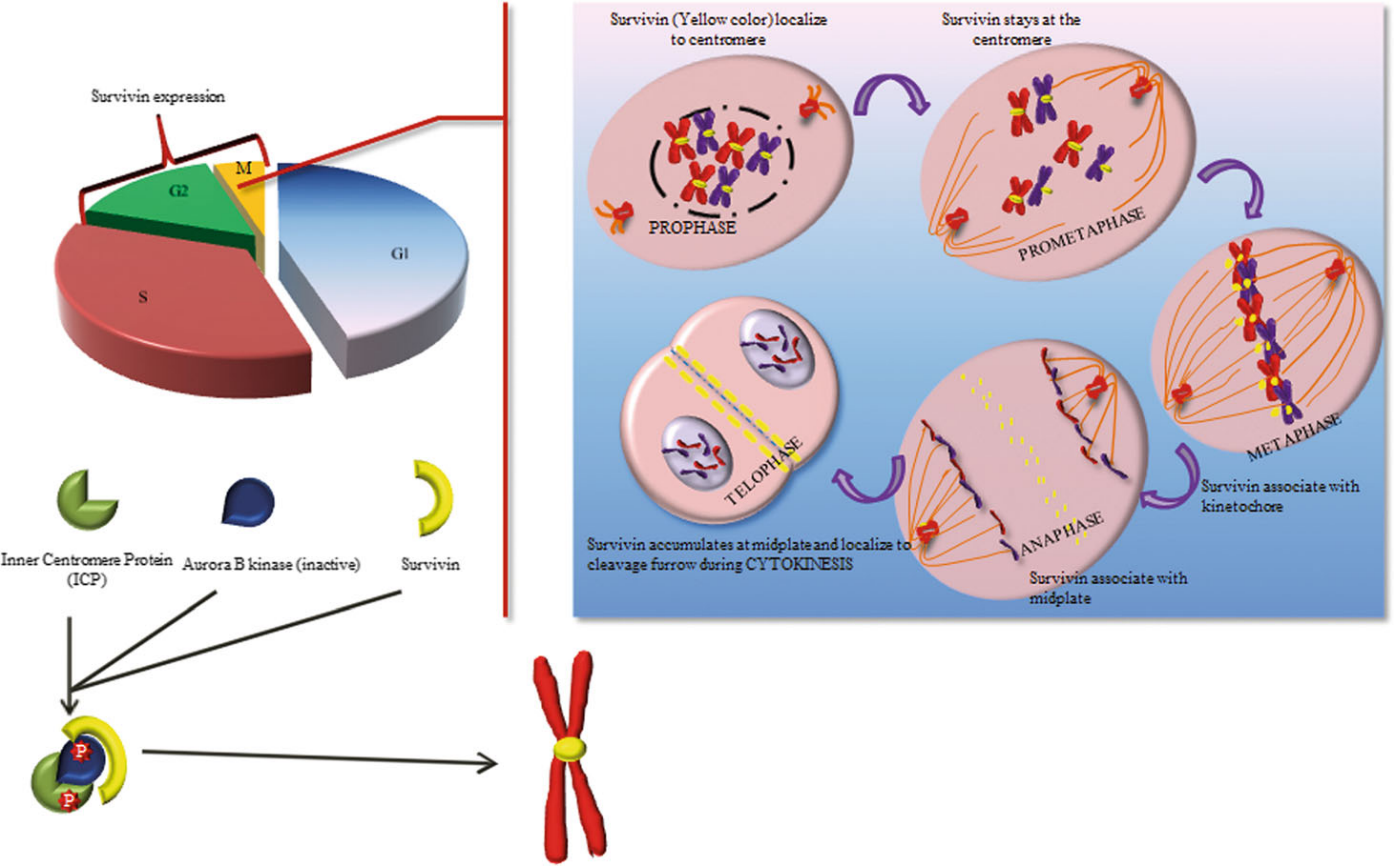
Role of survivin in cell cycle. In association with Aurora B and ICP, survivin forms a chromosomal passenger complex that bind to their target sites including centromere, midplate and cleavage furrow, where it regulates proper chromosome segregation and cytogenesis

Survivin plays an important role in the regulation of mitosis [30, 33]. It is expressed in a cell cycle dependent manner as reported mostly in G2-M phase [34]. During mitosis survivin interacts with tubulin and localizes to the mitotic spindle indicating its involvement in the regulation of mitosis [35]. It is now very well documented that survivin controls multiple facets of cell division in association with other proteins. It plays an important role in centrosome functions [21], microtubule assembly during metaphase and anaphase [36, 37], and spindle checkpoints (Fig. 1). Depletion of survivin causes defective cell division that involves activation of spindle checkpoints mediated by tumor suppressor protein p53 due to an arrest of DNA synthesis [30, 38]. Survivin-deficient cells frequently fail to complete both chromosome segregation and cytokinesis during mitosis. In the absence of survivin, sister chromatids start separating normally during anaphase, but often fail to move along with the main mass of segregating chromosomes that ultimately leads to an abnormal chromatid separation. Cytokinesis is also initiated normally, but in the absence of survivin it fails in late stages due to abnormality in spindle midzone and midbody microtubule formation [28, 39]. It has been demonstrated that these abnormalities in chromosome segregation and cytokinesis can be attributed to a defective CPC. Survivin forms a complex with Aurora B and inner centromere protein (ICP or INCENP) i.e., a characteristic of CPC [30, 35, 40]. This survivin/auroraB/ICP complex interacts with the central spindle midzone at the metaphase and anaphase chromosome, where it plays a crucial role in chromosomal segregation and cytokinesis [41] (Fig. 1). Aberrant mitosis and multi-nucleation has been observed in survivin-knockout cells [40, 42, 43]. Similar functions of survivin or its homolog have also been reported in other species. For example, in fission yeast, a survivin homolog Bir1P/Cut17P/Pbh1p forms a complex with Pic1P (an ICP homolog) and with replication initiation factor Psf2P, which regulates chromosomal segregation during mitosis [41].

### Role of survivin in apoptosis

**Fig. 2:**
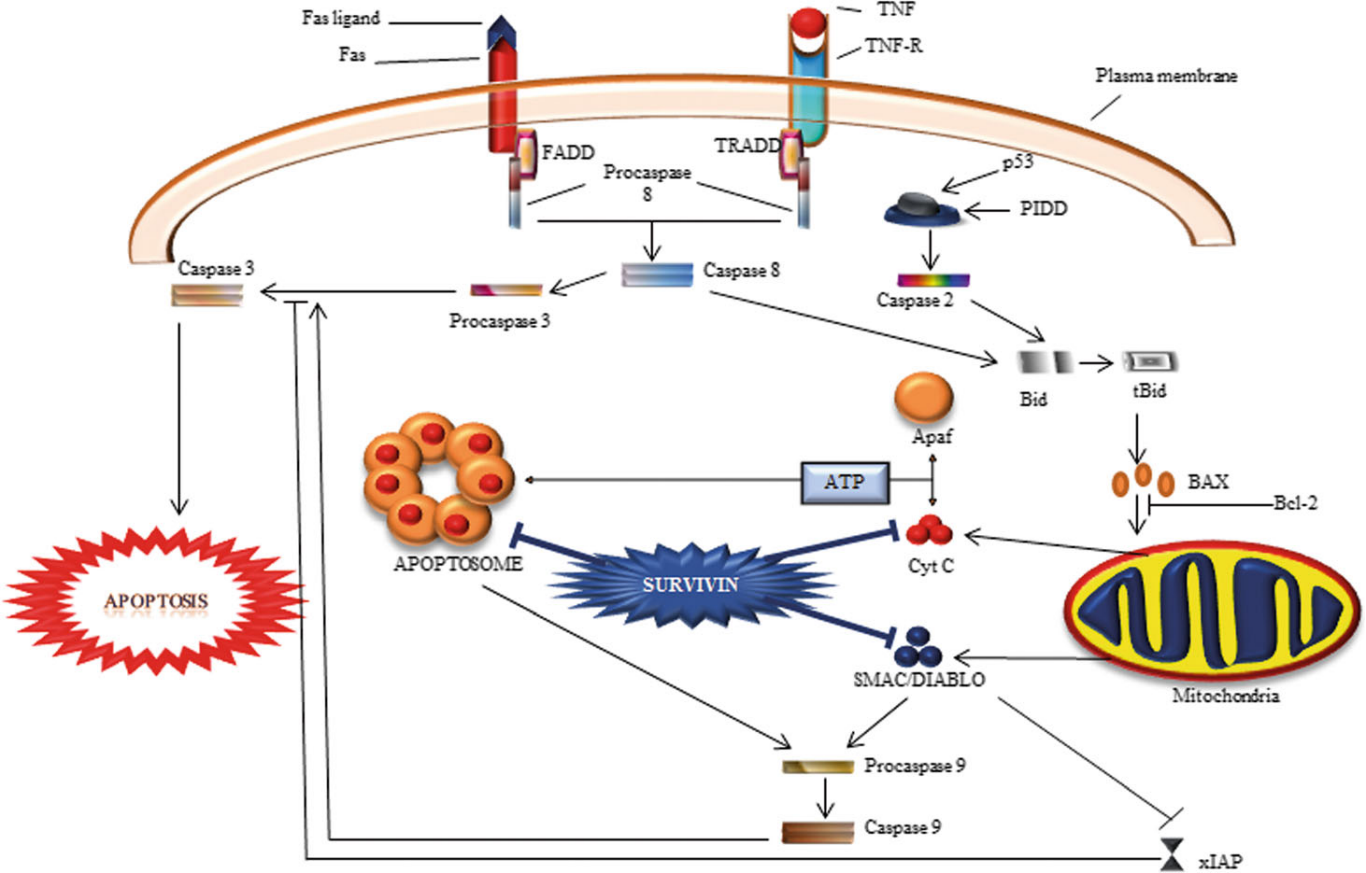
Role of survivin in apoptosis. Apoptosis can be initiated by the death-receptor (extrinsic) pathway or mitochondrial (intrinsic) pathway. Extrinsic pathway acts through caspase-8 and intrinsic pathway acts through caspase-9, but both pathways converge to activate the effector caspases-3,-7. Survivin largely interferes in mitochondrial-mediated apoptotic pathway. Apoptosome complex formed in association of Cyt-c, Apaf-1 and procaspase-9 in presence of dATP that leads to activation of procaspase-9. Survivin most probably blocks activation of caspase-9 by inhibiting apoptosome formation. It may also inhibit initiator caspase-9 and effector caspases-3 directly. Smac/DIABLO is a proapoptotic protein that inhibits activity of IAPs. Survivin antagonize the activity of Smac/DIABLO and may help in the action of another IAPs such as XIAP. XIAP is a strong inhibitor of apoptosis, which interacts directly with caspases and inhibits them

Apoptosis can be triggered with the two major types of stimuli, external and internal. The extrinsic apoptotic pathway initiates by the activation of death receptors (CD-95/Fas and TNFα receptors) through external signals following activation of initiator caspase-8 [35]. The intrinsic apoptotic pathway initiates due to intracellular signals that act through mitochondria. In response to signals, mitochondria release cytochrome-c (cyt-c) and Smac/DIABLO to form apoptosome for activating initiator caspase-9 [11]. Generally, mammalian IAPs inhibit apoptosis by direct or indirect inhibition of caspases [44] (Fig. 2).

In mammalian cells, mitochondria elicit arrays of cell death regulators [45]. Survivin is present abundantly in the inter-mitochondrial membrane space [46]. The mechanism(s) of survivin localization in mitochondria is currently unknown. However, molecular chaperone heat shock protein 90 (Hsp90) is thought to participate in importing many client proteins to mitochondria, and found to be associated with survivin and other IAPs [47, 48]. Interestingly, in normal cells survivin is not present in mitochondrial fractions [46], indicating that survivin translocation to mitochondria may be related to oncogenic transformation.

Several mechanisms have been proposed to explain anti-apoptotic activity of survivin. Some investigators have speculated that survivin may inhibit effector caspase-3 directly, even though it lacks a structural motif to bind directly with caspase-3, as is present in other IAPs. It has been shown that phosphorylation of survivin at threonine 34 (Thr34) is crucial for its anti-apoptotic functions. Another clue regarding anti-apoptotic mechanism of survivin came through its ability to interact with Smac/DIABLO suggesting that survivin may suppress activation of caspases indirectly. Smac/DIABLO acts like pro-apoptotic protein because of its participation in the formation of apoptosome and activation of caspase-9 [44]. Therefore, it is proposed that survivin most probably interferes in the down-stream steps of mitochondrial-apoptotic pathway, such as antagonizing apoptosome formation [12]. A point mutation in survivin protein at Asp-71 is sufficient to eliminate its interaction with Smac/DIABLO and anti-apoptotic function [49]. A strong inhibitor of apoptosis, XIAP interacts directly with caspases and inhibits them [49, 50]. It has been reported that Smac/DIABLO antagonizes functions of XIAP [50]. Therefore, presence of survivin may indirectly allow XIAP to function, ultimately leading to inhibition of apoptosis (Fig. 2).

### Regulation of survivin

Mechanisms of survivin regulation are still not fully understood. However, many signaling pathways and factors have been reported to activate survivin in cancer cells. It was originally thought that survivin up-regulation could be directly linked with cell proliferation, but its upregulation in non-proliferating Ki-67 MCF-7 breast cancer cells changed this concept [38]. It is now believed that overall intracellular pathways that activate survivin are more active in cancer as compared to normal cells. Inconsistent, reporter gene assays show negligible survivin promoter activity in normal cells as opposed to cancer cell lines [38], suggesting differences in regulation of survivin expression.

Several oncoproteins such as c-Myc and H-Ras that exceptionally expressed in malignant cells have been positively correlated with the upregulation of survivin. Studies show that oncoproteins, at least c-Myc and H-Ras induced survivin expression through PI3K signaling pathway, which is crucial for cancer cell survival [51, 52]. Amplification of survivin locus on 17q25 and demethylation of survivin exon 1 has been implicated in the upregulation of survivin in cancer cells [53, 54]. Importantly, mutations in retinoblastoma and p53 gene and its functional losses are often associated with the up-regulation of survivin in cancer cells. In normal cells, wild-type p53 and retinoblastoma directly or indirectly repress survivin transcription [55, 56]. Since, E2F activators can also induce survivin transcription, indicating that the retinoblastoma/E2F/p53 pathways may contribute to aberrant survivin expression. Activation of signal transducer and activator of transcription-3 (STAT-3) is associated with the up-regulation of survivin in gastric cancer, breast cancer, and primary effusion lymphoma [57–59]. In colorectal cancer, mutation in adenomatous polyposis coli (APC) tumor suppressor gene was associated with the aberrant stabilization of β-catenin and upregulation of survivin [60]. Nuclear factor-kappa B (NF-kB) is also associated with the transcriptional upregulation of survivin [61]. A p53/NF-κB crosstalk was reported to increase survivin expression in p53 mutant cells that shows strong chemoresistance [62, 63]. In myeloid leukemia, survivin expression is up-regulated in response to hematopoietic cytokines [64], suggesting that survivin expression can be controlled in autocrine or paracrine manner and hematopoietic cytokines may deliver their anti-apoptotic functions by increasing survivin. Besides upregulation, functional and structural stability of survivin in cancer cells requires post-translational modification in its interactions with other proteins. For example, survivin phosphorylation at threonine 34 by the cyclin dependent kinase 1 (CDK1) plays a crucial role in survivin function in cell division and in activation of pro-caspase 9 [65]. It has been recently shown that survivin functions is also controlled by acetylation at lysine residue K129, which directs survivin for nuclear localization [66, 67]. Survivin interacts with Hsp90, a central molecular chaperone to the cellular stress responses. This interaction involves the ATPase domain of Hsp90 and the BIR domain of survivin. Any disruption in this interaction induces proteasomal degradation of survivin [68, 69], suggesting Hsp90 protects survivin from degradation.

## Survivin expression in SCC: aggressiveness and poor prognosis

Growing number of publications are correlating survivin with negative tumor prognosis [17, 70, 71] Survivin is expressed in the vast majority of human cancers, including head and neck, laryngeal, esophageal, lung, breast, ovarian, gastric, central nervous system, colorectal, bladder, pancreatic, prostate, uterine, hepatocellular, and renal cancers, as well as melanoma and soft tissue sarcomas [72, 73]. Almost all SSCs express high level of survivin. Retrospective studies have been conducted to correlate expression of survivin with disease variables and clinical outcomes [72, 73]. Overexpression of survivin is often associated with tumor aggressiveness, poor prognosis, bad clinical outcome and overall low rate of survival in SCC patients (Table 1).

**Table 1:** Expression of survivin in SCCs. NC- no statistical correlation, IHC-immunohistochemistry, RT-PCR- reverse transcriptase, WB-western blotting

Type of SCC	Methods and number of samples	Correlation with survivin			Reference
		Clinicopathologic variables	Prognosis	Survival	
Oral and Oropharyngeal	IHC, WB (49)	Size, Nodal metastasis			[76]
	IHC (78)	Size, Aggressiveness, Invasion	Poor prognosis	↓	[77]
	IHC, RT-PCR, WB (110)	Early expression, Predictive invasive carcinoma			[227]
	IHC (13)	Distant non-lymphatic metastasis			[75]
	IHC, RT-PCR (71)	NC	Poor prognosis	↓	[78]
	IHC, WB (50)	Metastasis, Associated with Aurora B	Poor prognosis	↓	[81]
	IHC, RT-PCR (29)	Early expression, Predictive invasive carcinoma, Correlated with p53 expression			[74]
	PCR, WB	7,12-dimethylbenz[a]anthracene (DMBA) carcinogenesis			[80]
	Meta-analysis (1040)	Lymph node metastasis, Clinical stages	Poor prognosis		[79]
Laryngeal	IHC (68)	Site, Correlated with p53	Poor prognosis	↓	[70]
	IHC (86)	Metastasis			[228]
	IHC (102)	Metastasis			[229]
Esophageal	RT-PCR (51)	Nodal status	Poor prognosis	↓	[83]
	RT-PCR (57)	Metastasis	Poor prognosis	↓	[84]
	IHC (84)		Poor prognosis	↓	[86]
	Meta-analysis (610)	Lymph node Metastasis	Poor prognosis	↓	[71]
Non-small cell lung cancer	IHC (58),	NC	Poor prognosis	↓	[95]
	IHC, RT-PCR (83)	Early marker, Tumor stages			[91]
	Meta-analysis (2703)	Tumor stages	Poor prognosis		[230]
	RT-PCR (71)	NC		↓	[90]
	IHC (102)	Tumor size, Distant metastasis	Poor prognosis	↓	[92]
	RT-PCR (140)	Tumor differentiation, Aggressiveness, Correlated with p53 mutation			[93]
Skin	IHC, WB (89)	Size, Nodal metastasis		↓	[76]
	IHC (47 different groups)	Early marker, Disease progression			[105]
	IHC (62 different groups)	Keratinocytic neoplasms, Hyperproliferative lesions			[104]
Cervical	IHC (17)	NC			[231]
	IHC, WB (53)	Size, Lymphovascular invasion	Poor prognosis		[111]
	IHC (59)	Size, Tumor grade, Clinical stages	Poor prognosis		[112]
	IHC (73)	HPV			[114]
	RT-PCR (50)	Tumor stages, Correlated with bcl2			[109]
	IHC (50)	Clinical stages, CIN grade, Lymph node metastases, Correlated with p16INK4A	Poor prognosis		[110]
	IHC (49)	Clinical stage, Tumor size, Lymph node metastasis, Correlated with PTEN	Early diagnostic and poor prognosis		[232]

### Head and neck SCC

We have reported that survivin is overexpressed in majority of OSCC tissues, and in ~50% premalignant tissues [74], pointing out its early involvement in OSCC progression. As reported, accumulation of mutated p53 is considered a factor for survivin up-regulation [55, 56], we observed a positive correlation between survivin and p53 expression in premalignant and malignant OSCC tissues [74]. Studies have established a correlation between survivin status and oral cancer aggressiveness [75]. For examples, survivin significantly segregated with high-grade and undifferentiated tumors and invariably associated with lymph node metastasis (indicators of tumor aggressiveness) [76, 77], and ultimately this affects patient’s survival rate. These findings suggest that the cases of OSCC with more aggressive and invasive phenotype may identify on the basis of survivin expression, and therefore, could influence the decision for the therapy at the time of diagnosis. As compared to cytoplasmic survivin, lower nuclear expression of survivin has been shown a strong predictor for relapse-free survival in the oral cancer patients [78], suggested survivin as an early predictive marker for disease outcome. A meta-analysis study from 15 published articles (1040 cases), in which survivin expression was determined either by immunohistochemistry or RT-PCR in OSCC, found a positive correlation between survivin expression and lymph node metastasis and clinical stage [79]. However, analysis did not find an association between survivin expression and tumor differentiation grade, and depth of invasion. Other meta-analysis study from 610 esophageal cancer patients revealed a significant correlation between survivin over expression and poor overall survival [71].

By using hamster buccal-pouch mucosa experimental model for oral carcinogenesis, Dr. Chen and colleague found survivin up-regulation in all 7,12-dimethylbenz[a]anthracene (DMBA)-induced hamster buccal-pouch squamous-cell carcinomas. They also found demethylation of survivin allele in DMBS-induced OSCC, suggested gene expression may be modulated by an epigenetic mechanism [80]. As survivin regulates cell division, a positive correlation has been observed between survivin expression other chromosomal passenger proteins, such as Ki-67 and Aurora-B expression [81], which involves in chromosome segregation. Tumors with increased nuclear survivin and Aurora-B expression exhibited marked malignant behaviors [81]. Dr. Keller and colleagues were able to distinguish between human papilloma virus (HPV) positive vs. negative HNSCC samples on the basis of survivin level [82]. The results show that HPV-negative tumors have high level of survivin and poorer prognosis than HPV-positive HNSCC. Tumors with less than a median level of survivin expression were associated with improved patient survival as compared to tumors with more than a median level of survivin [82], proving survivin as a marker for improved survival.

### Esophageal SCC

ESCC is one of the most malignant tumors. Survivin is overexpressed in most of the esophageal cancer [83]. Malignant tissues showed significantly higher level of survivin as compared to non-malignant tumors [83, 84]. The different splice variants of survivin were found to be associated with diverse tumor clinicopathological variables. For example, a high cytoplasmic survivin correlated with histological differentiation and invasion, and a high survivin 2B splice variant was associated with poor prognosis in esophageal cancer patients [85]. High nuclear level of survivin also correlated with poor prognosis [86]. In esophageal cancer, survivin overexpression provides a resistant phenotype, as indicated by increased rate of tumor recurrence and lower patient survival in the case of high expression of survivin as compared to low survivin expression [83, 84, 86]. There is an inverse correlation between miR-214-3p and survivin expression with the re-expression of miR-214-3p down-regulate survivin expression via RNA-binding protein (RBP) CUG-BP1 leading to reduction of chemotherapy resistance in ESCC [87]. Case controls studies from different populations showed that single nucleotide polymorphisms in survivin gene, for example -31G/C influence the susceptibility to esophageal cancers in Indian [88] and Chinese population [89].

### Lung SCC

Non-small cell lung carcinoma (NSCLC) is a major class of lung cancer. Most of the NSCLC (80-90%) shows overexpression of survivin [90, 91]. Studies show a significantly higher level of survivin in SCC as compared to adenocarcinoma of lung, [92–94], where it contributes to poor prognosis and decreased patient survival [90, 95]. Higher nuclear survivin has been identified as an independent prognostic factor for lung SCC [95]. In lung SCC, survivin up-regulation is associated with increased tumor angiogenesis and metastasis [96]. Study suggested an early detection of survivin can be considered as useful diagnostic tool for the detection of lymph node micrometastasis for stage I NSCLC patients [97]. Since p53 is a regulator for survivin, mutation in p53 gene has been positively correlated with the up-regulation of survivin in lung SCC [93]. In addition, polymorphisms in the survivin gene have been found to influence survivin production and thereby modulate susceptibility to lung cancer [98, 99].

### Skin SCC

Squamous cell carcinoma (SCC), basal cell carcinoma (BCC) and melanoma are three major type of skin cancers. BCC and SCC are sometime called non-melanoma cancer [100]. Survivin is overexpressed in both melanoma and non-melanoma skin cancers [76, 101–103]. It is overexpressed in 64-92% skin SCC compared to normal skin [76, 104]. The level of survivin is more in high grade and undifferentiated tumors with lymph node metastasis indicating tumor aggressiveness and invasive behavior [76, 105]. The expression of survivin was also present in high percentage of premalignant lesions of Bowen’s disease (SCC in situ) and hypertrophic actinic keratosis (HAK), suggesting that its appearance occurs early during keratinocyte transformation [105]. Dallaglio et al. [103] analyzed intracellular localization of survivin and its correlation with keratinocytes differentiation and SCC. They found marked increases of nuclear survivin (not cytoplasmic) in actinic keratosis and in SCC in situ, and that was highest in poorly differentiated SCC. They found survivin mostly localizes in the deep infiltrating areas of tumors that associated with increased cell migration [103]. In skin, it is quite established that genetic alterations in keratinocyte stem cells (KSC) gives rise to SCC-derived Stem-like Cells (SCC-SC) [106]. Survivin overexpression is a key factor in the transformation of KSC to SCC-SC, and tumor-producing KSC can be isolated on the basis of survivin expression [107]. Since, survivin support maintaining SCC cancer stem cells [101, 108], it is one of the key factor for tumor recurrence and poor clinical outcome in skin cancer

### Cervical SCC

Many studies have found survivin overexpression in cervical SCC as compared to the normal tissues [109–112], and its expression associated positively with lesion size, lymphovascular invasion and poor prognosis [111], tumor grade and clinical stages [110, 112]. HPV infection is a leading risk factor for the development cervical cancer [113]. Studies found a positive correlation between survivin expression and HPV infection in cervical carcinoma [113, 114]. In contrary to other SCCs (in which nuclear survivin is associated with poor prognosis), cytoplasmic survivin expression is associated with poor prognosis in cervical carcinoma [115]. All these findings suggest survivin participate in the onset and progression of cervical carcinoma.

### Diagnostic potential

Commonly used techniques, such as ELISA and immunohistochemistry are able to measure survivin in tumor samples. In fact, many pharmaceutical companies, such as Cell Signaling (Cat. 7169), Novus Biologicals (Cat. BEK-2121-2P) have come up with commercial kits for survivin detection in biological samples. This may be a quick test for poor prognosis and identifying patients with high risk of tumor recurrence, and could be useful in decision making by clinicians on whether such these patients should be subjected aggressive alternative protocols. Several clinical trials are being conducted to establish survivin detection assays for cancer diagnosis (Table 2).

**Table 2:** Clinical trials of survivin-targeting therapies

Identifier or Reference	Sponsors	Condition	Purpose	Intervention	1) Primary 2) secondary outcome measures	Phase/status and outcome	First received	Last updated/closed
Survivin-targeting immunotherapies and gene therapy								
UMIN000000976	University Hospital Medical Information Network	Oral cancer	Study to evaluate the safety and the efficacy of survivin-2B80-88 peptide vaccination in HLA-A24-positive patients with advanced or recurrent oral cancer.	Biological: Survivin-2B80-88 peptide vaccination	1) Safety 2) Efficacy	Phase 1/Completed: survivin-2B peptide vaccination was safe and had therapeutic potential for oral cancer patients	Sept 1, 2003	Feb 01, 2011
NCT01250470	Roswell Park Cancer Institute	Malignant glioma	Study the side effects of survivin peptide vaccine therapy when given together with sargramostim in treating patients with malignant glioma.	Other: Laboratory Biomarker Analysis Drug: Montanide ISA-51/Survivin Peptide Vaccine Biological: Sargramostim	1) Safety and toxicity 2) Immune response	Phase I/Completed	Nov 24, 2010	Feb 24, 2017
NCT02851056	H. Lee Moffitt Cancer Center and Research Institute	Multiple Myeloma	Test the safety and immune responses of a new survivin vaccine and its effects on multiple myeloma cancer, when administered before and after their autologous hematopoietic cell transplant (HCT). The name of the vaccine is called Dendritic Cell Survivin Vaccine (DC: AdmS)	Biological: Survivin Vaccine Procedure: Autologous Hematopoietic Cell Transplantation Biological: Prevnar 13 Drug: Granulocyte-colony Stimulating Factor	1) Safety of DC: AdmS when administered to patients with myeloma before and at day +21 after autologous hematopoietic stem cell transplant. 2) The ability of DC: AdmS to induce T cell immune responses against survivin when administered to patients with myeloma	Recruiting	July 28, 2016	Dec 7, 2016
NCT00108875		Malignant Melanoma Pancreatic Cancer, Colon, Cancer, Cervical Cancer	Evaluates the safety, the immunological response and the clinical outcome of a vaccination with survivin peptides for patients with advanced melanoma, pancreatic, colon and cervical carcinoma.	Biological: Survivin peptide vaccine	1) Progression-free survival, Overall survival, Immunological response 2) Best response	Phase 1 Phase 2/Unknown	April 19, 2005	July 27, 2006
NCT00961844	Oslo University Hospital	Metastatic Malignant Melanoma	Study the safety and effectiveness of chemotherapy with immunotherapy by giving the patients Temozolomide, before vaccination. The investigators have also included hTERT and survivin mRNA in the vaccine. Finally, the investigators want to introduce ex vivo T cell expansion after lymphodepletion for the patients who show an immune response.	Biological: Dendritic cells - transfected with hTERT-, survivin- and tumor cell derived mRNA + ex vivo T cell expansion and reinfusion Drug: Temozolomide	1) Safety and toxicity of vaccination with DC transfected h-TERT mRNA, survivin mRNA and tumor cell mRNA. 2) Evaluation of immunological responses, time to disease progression and survival time	Phase 1 Phase 2/Terminated	Aug 12, 2009	Aug 2014
NCT00573495	University of Pennsylvania	Breast Cancer	Study on how to activate the immune system with a vaccine, which made up of two proteins found in breast cancer: telomerase and survivin.	Biological: hTERT/Survivin Multi-Peptide Vaccine	1) Safety 2) Immunologic response	Phase 1/Completed	Dec 12, 2007	Sept 27, 2016
NCT00074230	University Hospital Erlangen	Melanoma (Skin)	Study the effectiveness of vaccine therapy using autologous dendritic cells with antigens in treating patients with stage IV cutaneous melanoma.	Biological: Autologous Dendritic Cells loaded with MAGE-A3, MelanA and Survivin	1) Safety and tolerability, overall survival. 2) Immune response and disease progression	Phase 1 Phase 2/Completed	Dec 10, 2003	May 11, 2015
NCT02323230	ImmunoVaccine Technologies, Inc.	Diffuse Large B-Cell Lymphoma	Assess the efficacy and safety of DPX-Survivac plus low dose cyclophosphamide in subjects with recurrent diffuse large B-cell lymphoma (DLBCL) who are not eligible for transplant.	Biological: DPX-Survivac Drug: Cyclophosphamide	1) Objective response rate 2) Immune response and levels of cell mediated immunity targeting the survivin epitopes	Phase 2/Recruiting	Dec 12, 2014	Dec 14, 2015
NCT01416038	ImmunoVaccine Technologies, Inc.	Ovarian Cancer Fallopian Tube Cancer Peritoneal Cancer	Determine the safety and immunogenicity profiles of DPX-Survivac, a therapeutic vaccine co-administered with a regimen of low dose oral cyclophosphamide.	Biological: DPX-Survivac Drug: low dose cyclophosphamide (oral)	1) Number of reported adverse events and Progression free survival 2) Levels of cell mediated immunity targeting the survivin epitopes	Phase 1 Phase 2	Aug 9, 2011	Dec 14, 2015
NCT02688673, NCT02693236, NCT01924156	Affiliated Hospital to Academy of Military Medical Sciences	Small- Cell Lung Cancer, Esophagus Cancer, Renal Cell Carcinoma	Evaluate the safety and efficacy of dendritic cells (DC) combined with cytokine-induced killer (CIK) cells to treat cancer patients	Biological: adenovirus-transfected autologous DC vaccine plus CIK cells	1) Objective rate response (CR + PR) as measured by RECIST criteria 2) Number of participants with adverse events	Phase 1 Phase 2/Ongoing	2013-2016	2016
Survivin-targeting small molecule therapies								
NCT00537121	Roswell Park Cancer Institute	Esophageal Cancer, Gastric Cancer, Liver Cancer	Study the side effects and best dose of vorinostat (SAHA) when given together with irinotecan, fluorouracil, and leucovorin in treating patients with advanced upper gastrointestinal cancer. SAHA suppresses tumor cells growth by blocking HDAC that also involve inhibition survivin and TGF-beta signaling.	Drug: fluorouracil Drug: irinotecan hydrochloride Drug: leucovorin calcium Drug: vorinostat Other: pharmacological study	1) Maximum tolerated dose (MTD) of vorinostat (SAHA) when administered continuously and intermittently with standard doses of irinotecan hydrochloride, fluorouracil, and leucovorin calcium (FOLFIRI). Recommended phase II dose (RPTD) of SAHA when administered continuously and intermittently with standard doses of FOLFIRI 2) Toxicity of the SAHA and FOLFIRI combination. Effects of SAHA and FOLFIRI combination on TGF-β signaling and survivin expression. Response rate. Progression-free survival Overall survival	Phase 1/Completed	Sept 27, 2007	June 26, 2013
NCT01398462	JW Pharmaceutical	Acute Myeloid Leukemia, Chronic Myelomonocytic Leukemia, Myelodysplastic Syndrome Myelofibrosis	Test safety, efficacy, and antitumor activity of CWP232291. This drug targets beta-catenin for degradation and thereby inhibits the expression of cell cycle and anti-apoptotic genes such as cyclin D1 and survivin	Drug: CWP232291	1) To determine Maximum Tolerated Dose (MTD)and dose limiting toxicities (DLTs) 2) Pharmacokinetics, and assess the anti-tumor activity	Phase 1	July 17, 2011	March 7, 2016
NCT00664586	Erimos Pharmaceuticals	Refractory Solid Tumors Lymphoma	Continuous infusion study designed to explore if constant concentration over time adds to the effectiveness of terameprocol without increasing toxicity. It will also explore weekly dosing as an option.	Drug: Terameprocol (EM-1421)	1) To determine Maximum Tolerated Dose (MTD)and dose limiting toxicities (DLTs) 2) Assess the anti-tumor activity	Phase 1/Terminated due to funding constraints	April 21, 2008	Feb 20, 2016
NCT00664677	Erimos Pharmaceuticals	Leukemias: AML, ALL, ATL, CML-BP, CLL, MDS, CMML	Determine the safety, maximum tolerated dose,dose limiting toxicity of Terameprocol and determine the pharmacokinetics of Terameprocol given as intravenous infusion.	Drug: Terameprocol (EM-1421)	1) To determine Maximum Tolerated Dose (MTD)and dose limiting toxicities (DLTs) 2) Pharmacokinetics and assess the anti-tumor activity	Phase 1/Terminated due to funding constraints	April 21, 2008	Feb 20, 2016
[223]	H. Lee Moffitt Cancer Center	Advanced solid malignancies or lymphoma	Determine the maximum-tolerated dose (MTD) and assess the safety, pharmacokinetics, and preliminary evidence of antitumor activity	Drug: YM155 (First human trial)	1) To determine Maximum Tolerated Dose (MTD) and dose limiting toxicities (DLTs) 2) Pharmacokinetics of drug and preliminary evidence of 2anticancer activity	Phase 1/Completed: Drug is safe to administer with no severe toxicities, and showed antitumor activity		Nov 10, 2008
[224]	National Cancer Institute	Advanced non–small-cell lung cancer (NSCLC).	Evaluate the antitumor activity and safety of YM155, a novel, small-molecule suppressor of survivin, as single-agent therapy.	Drug: YM155	1) Safety and tolerance 2) Anti-tumor activity	Phase 2/Completed: Drug showed modest single-agent anti-tumor activity, and a favorable safety/tolerability profile was reported		Sept 20, 2009
[226]	Georgetown University Hospital	Refractory diffuse large B-cell lymphoma	Study toxicity and efficacy YM155	Drug: YM155	1) Safety and tolerance 2) Anti-tumor activity	Phase 2/Completed: Drug was well tolerated and showed limited anti-tumor activity as a single agent		June 15, 2012
NCT00514267	Astellas Pharma Inc	Prostate Cancer Tumors	Determine the feasibility and safety of administering YM155 in combination with docetaxel	Drug: YM 155 Drug: Docetaxel Drug: Prednisone	1) Occurrence of dose limiting toxicities 2) Assessment of safety, efficacy and pharmacokinetics	Phase 1 Phase 2/Completed	Aug 7, 2007	July 23, 2015
NCT01100931	National Cancer Institute	NSCLC Solid Tumors	Determine the efficacy of the combination of carboplatin, paclitaxel, and YM155 in the treatment of non-small-cell lung cancer	Drug: YM155 Drug: Carboplatin Drug: Paclitaxel	1) Assessment of safety, efficacy and pharmacokinetics 2) Anti-tumor activity	Phase 1 Phase 2/Completed	April 8, 2010	Sept 29, 2015
Diagnostic								
NCT00315653	Fujirebio Diagnostics, Inc.	Bladder Cancer	Evaluate the ability of urinary Survivin mRNA measurement to estimate the risk of bladder cancer at the time of cystoscopy in subjects with no prior history of bladder cancer presenting with microscopic or macroscopic hematuria	Procedure: Urine Sampling	Evaluation of the Survivin Urine mRNA Assay	Completed	April 18, 2006	March 12, 2008
NCT02016833	PX Biosolutions	Ovarian Serous Adenocarcinoma, Undifferentiated Carcinoma of Ovary, Cervical Cancer, Cervical Intraepithelial Neoplasia, Grade 3 Acute Myeloid Leukemia, Chronic Myeloid Leukemia	Establishing immunological assays for the qualitative and quantitative evaluation of WT-1, Survivin and HPV16 E7-specific immune responses in cancer patients	Procedure: Blood Sampling	Development and validation of ELISpot and tetramer assays	Completed	Dec 5, 2013	April 29, 2015

Survivin or its auto-antibodies have been found in biological fluids of cancer patients that may be used for cancer diagnosis. For example, sensitive diagnostic tests have been developed that are based on the presence of survivin in urine of bladder cancer patients [116, 117]. Similarly, presence of survivin auto-antibodies in the saliva of OSCC patients provides a novel and practicable approach for OSCC screening [118, 119]. In addition, anti-survivin antibodies were also detected in the circulating blood of cancer patients [120], which can be exploited as a cancer diagnostic tool.

## Therapeutic targeting of survivin

Survivin has been known for regulating various cellular processes including cell growth and apoptosis. Expression of survivin is a very consistent feature of hyper-proliferative lesions, which contribute in the development of hyperplasia. Several techniques have been developed to examine therapeutic potential of survivin in cancer treatment. These include inhibition of survivin expression using antisense oligonucleotides, ribozyme, small interfering RNA (siRNA) or short-hairpin RNA (shRNA) techniques or antagonizing survivin function by dominant-negative survivin or by small molecules. Therapeutic uses of survivin have been evaluated in several preclinical and clinical studies.

### Antisense technology

**Fig. 3:**
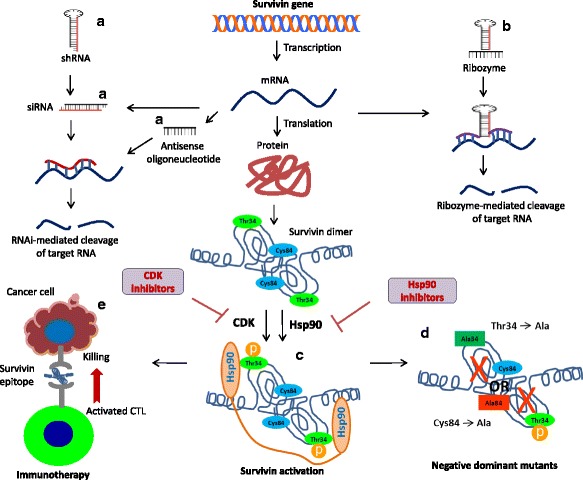
Schematic representation of different techniques to target survivin for therapeutic purposes. **a** Antisense technology, such as antisense oligonucleotides, siRNA and shRNA target survivin mRNA to inhibit translation. **b** Ribozyme is also an advanced antisense method to target mRNA. The specificity of ribozyme determined by the paired regions flanking the cleavage site. **c** Dimerization and phosphorylation on Thr34 residue is essential for survivin activation and Hsp90 provide stability to survivin dimer. Small molecule antagonists for survivin activation, such as CDK and Hsp90 inhibitors, able to inhibit survivin phosphorylation or its interaction with Hsp90, consequently inhibit survivin functions. **d** In dominant negative mutants, an essential amino acid of the survivin is replaced by another amino acid that leads to the loss of function. For example, Thr34Ala mutant inhibit survivin activation through abolishing phosphorylation of Thr34 residue, and Cys84Ala mutant inhibit survivin dimerization. **e** Survivin-directed immunotherapy approaches. Peptides-derived from survivin can induce CTL activity against tumor cells

Antisense technologies are proving a useful tool for cancer therapeutics. These include uses of antisense oligos, siRNA and shRNA techniques, which specifically suppressed expression of target genes (Fig. 3). Antisense-RNAs can be expressed directly in cells by delivering a plasmid or viral vectors or it can be synthesized chemically and transfected into cells. Since survivin is overexpressed in many cancers, its down-regulation by antisense-oligos could be of therapeutic use. Indeed, anti-survivin oligos have been evaluated in many cancers to suppress survivin and its effects on cell death. Deliveries of these oligos in cancer cells induce apoptosis and also increase anti-cancer effects of other therapies such as chemotherapy and radiotherapy. Dr. Olie and colleagues [121] have tested many anti-survivin oligonucleotides in study on NSCLC [121]. Out of many designed oligonucleotides, 4003 was reported to be most effective in suppressing NSCLC growth. We are conducting studies to investigate the role of survivin in HNSCC resistance to conventional drugs. Our results have shown that survivin overexpression in HNSCC cells provide resistance against conventional drugs. siRNA-mediated suppression of survivin significantly inhibits HNSCC cell proliferation and also increases response of chemotherapy and radiotherapy [122, 123]. For pre-clinical studies, we have developed lentivirus vector to deliver survivin-siRNA [124]. A significant growth reduction was observed in human HNSCC tumor xenograft in mouse model with survivin knockdown-using lentivirus-siRNA therapy [125]. Furthermore, a high efficacy was observed when we used a combination of lentivirus-siRNA and chemotherapy or radiotherapy. Many other in vitro and in vivo studies have been conducted in which anti-survivin techniques were used either alone or in combination with conventional drugs to control cancer cell proliferation including most of the SCCs, such as oral [126], laryngeal [127, 128], head and neck [129], skin [103], esophageal [87, 130], and lung [131]. Wen et al. [132] investigated inhibitory function of survivin in laryngeal SCC cell lines GRIM-19 and Hep-2 using plasmid-based survivin-specific shRNA. During proliferation of laryngeal cancer cell lines undergoing transfection with p-siRNA, survivin was markedly inhibited (79%). In vivo study also showed a significant suppression of Hep-2 tumor growth and apoptosis induction due siRNA-mediated silencing of survivin. Survivin inhibition by shRNA abrogated radiation-induced G2 phase arrest and amplified radiation-induced apoptosis [128]. Stoleriu et al. [133] successfully tested multimodality therapy regimen to treat chemoresistant NSCLC cell lines. siRNA-mediated knockdown of survivin along with other genes in these cell lines sensitized them to chemotherapies and significantly induced apoptosis. Over-expression of survivin in OSCC makes it a potential gene therapy target. A lentivirus vector encoding shRNA targeting survivin was used to suppress the survivin gene in a study [134]. Inhibition of survivin reduced proliferation of tumor cells in vitro and sensitized cells to radiation and vincristine. In the OSCC xenograft model, both tumor development and growth of established tumors was inhibited with survivin-lentivirus therapy. Similar growth inhibitory effects of survivin have been observed by using anti-survivin siRNA in esophageal [87, 130] and skin SCC tumor xenograft models [103].

As in SCCs, inhibition of survivin showed anti-proliferative effects in many other malignancies. An antisense oligo that targets 232 to 251 nucleotide sequence of survivin showed a significant killing of mesothelioma cancer cells and also sensitized them to chemo-radiotherapy [135]. Growth of lymphoma cells was also arrested by using anti-survivin oligos [136]. In a study, adenoviral antisense vector targeting survivin (pAd-CMV-SAS) was used to treat colon cancer, which resulted in an cell cycle arrest of cancer cells in Go/G1 phase and induced chemotherapy-mediated cells death [137]. PC-3 prostate cells showed nuclear fragmentation, hypodiploidy, activation of caspase-3, all apoptotic signatures when treated with antisense survivin cDNA [138]. Similar observations were made in human neuroblastoma cells [139]. Survivin knockdown by adenoviral vector (Adv-siSurv) expressing multiple anti-survivin oligos induced apoptosis in many different cancer cell lines, in vitro [140, 141]. Intratumoral injection of this adeno-vector in human tumor xenograft mouse model significantly suppressed tumor growth. A shRNA containing two reverse repeat motifs was designed to target survivin gene. Treatment of liver cancer cell lines with this vector efficiently down-regulated expression of survivin and induced apoptosis [142]. These observations suggest that antisense technology targeting of survivin could be a potential selective cancer therapy.

### Dominant negative mutants

In this technique, a nonfunctional protein is formed due to the replacement of an essential amino acid by another amino acid. Due to having the same targets, these nonfunctional proteins compete with normal protein and dilute its function. Quite few dominant negative mutants have been designed and tested for the inhibition of survivin, out of which T34A mutant is well studied, in which threonine is replaced with alanine at amino acid 34 (Fig. 3). Transduction of many different cancer cell types including lung, breast, cervical, prostate, colorectal, liver, and skin with adenovirus vector (pAd-T34A) encoding a non-phosphorylated T34A mutant of survivin, induced apoptosis by increasing cyt-c release from mitochondria and activation of procaspase-3. This treatment also sensitized cancer cells to chemotherapeutic agents such as taxol and adriamycin [143]. Transfection of malignant HeLa cells with T34A survivin mutant could reverse the malignant phenotype [144]. Intratumor injection of pAd-T34A in a tumor bearing mouse significantly suppressed pre-established tumor size by inducing apoptotic cell death [143]. Injection of pAd-T34A in peritoneal cavity significantly reduced tumor growth of breast cancer cells in immunodeficient mice. Similar results were observed in mice bearing NSCLC tumor [145]. Interestingly this treatment did not cause any visible effects on normal cell viability and systemic toxicity. In a similar in vivo study, intratumoral injection of pAd-T34A suppressed tumor growth in prostate cancer mouse model [146] and enhanced anti-androgen sensitivity [147]. These results suggest that the uses of pAd-T34A may selectively target tumor cells with very limited normal tissue toxicity.

Another interesting survivin-mutant (C84A) was constructed by replacement of cystein residue at amino acid 84 with alanine. In gastric cancer cell lines, transfection with plasmid expressing C84A mutant decreased cell growth and induced spontaneous apoptosis [148]. In prostate cancer cell lines, C84A treatment is able to induce all apoptotic hallmarks including hypoploid DNA, activation of caspases, and cleavage of caspase substrates such as Poly (ADP-ribose) polymerase. Similar results were obtained with cutaneous SCC, where C84A transfection resulted in spontaneous apoptosis even without adding any other genotoxic stimuli. Due to this treatment, cells were arrested in sub-G0/G1 phase that correspond to apoptosis with a 4 N DNA content [105]. Injection of adeno-associated virus in colon cancer mice model encoding C84A mutant suppressed angiogenesis and tumor growth without causing normal cell toxicity [149]. In large-cell lymphomas, injection of survivin mutant C84A reduced tumor cell growth and enhanced cell death by increase of tumor-specific cytotoxic T lymphocytes [150] (Fig. 3).

Interestingly combined therapy using survivin negative dominant mutants and chemo-radiotherapies showed better anti-tumor effects. Mostly the effects of survivin mutants combined with other drugs were reported to be synergistic (more than additive). For instance, combined treatment of NSCLC cells with T34A and radiation induced more cell death than single drug treatments, and this effect was more than additive, suggesting that the inhibition of survivin by T34A mutant could sensitize NSCLC cells to radiation treatment [145]. In another study, lyposome complex was used to deliver survivin T34A mutant with or without cisplatin. An intravenous injection of lyposome-T34A in mice, tumor volume was reduced significantly. The antitumor effect of lyposome-T34A combination with cisplatin was greater than their anticipated additive effects, suggesting a synergistic interaction. In vivo studies also showed anti-angiogenesis effects of survivin dominant negative constructs [151]. Zhang et al. [152] constructed a double dominant negative mutant of survivin (T34A-C84A) for understanding whether it could have better potential to kill cancer cells. Treatment of hepatocellular cancer cells with the adenoviruses expressing this double mutant (Ad-T34A-C84A) showed much stronger cell killing as compared to single survivin mutants T34A or C84A alone [152].

### Ribozyme technique

Ribozyme (ribonucleic acid enzyme) is a new approach to degrade RNA in cells for therapeutic purposes [153]. Several ribozyme molecules have been developed, of which hammerhead ribozyme is best studied [154]. Hammerhead is the smallest ribozyme containing a highly conserved core residue required for RNA cleavage and three base-paired stems required for identifying the target site. It cleaved target mRNA just after NUH sequence (N can be any nucleotide, and H can be any nucleotide except G) [155]. The specificity of target mRNA cleavage by ribozyme is much higher than siRNA approaches, which often produce off-target effects. Paired flanking regions of cleavage site determine the specificity of cleavage (Fig. 3).

Several ribozymes have developed to inhibit survivin in cancer cells. Choi et al. [156] have designed two hammerhead ribozymes (RZ1and RZ2) to target human survivin mRNA. These ribozymes cleaved survivin mRNA at nucleotide positions +279 and +289. For functional study, an MCF-7 breast cancer cell line was transduced with adenoviral vector encoding these ribozymes, which significantly reduced expression of survivin, and consequently induced apoptosis[156]. In prostate cancer cell lines (PC-3 and DU145), infection with adenoviral vector encoding a ribozyme targeting 3’-end of the survivin-mRNA induced apoptosis [157]. Transduction of melanoma cancer cells with adenovirus-vector expressing ribozyme increased sensitivity of cancer cells to chemo-radiotherapy treatment [157, 158]. Four hammerhead ribozymes (R1 to R4) to suppress survivin gene were designed by Fei et al. [159]. Adenoviruses encoding these ribozymes have been tested in vitro and in vivo for controlling cancer cell growth. Results showed that inhibition of survivin deregulates mitotic cell division and induces caspase-3-dependent apoptosis of cancer cells. Injection of these ribozyme adenoviruses also suppressed tumor growth in a xenograft mouse model of hepatocellular carcinoma. Study further demonstrated that the combination of these ribozymes can give even better clinical outcomes, as reflected by higher cancer cell death in a combination treatment of R1, R3 and R4 as compared to single ribozyme treatment. All forms of survivin were cleaved during this combination treatment, which gives a very strong anti-cancer effect [159]. Despite high substrate cleavage efficiency, clinical application of ribozyme is still limited due to misfolding and RNA degradation of ribozyme, when fused to a carrier. An innovative chimeric ribozyme was constructed by Liu et al. (2007). This chimeric ribozyme derived from a motor pRNA of phi29 bacteriophage, shows enhanced stability and robustness in folding [160]. In a variety of in vitro and in vivo cancer models, treatment with this chimeric ribozyme has been found to suppress survivin mRNA and protein efficiently, and strongly induced apoptosis with very limited normal cell cytotoxicity. These studies clearly indicate that ribozyme-targeting of survivin in cancer cells could be of therapeutic use.

### Immunotherapy

Enhancing immune response against cancer cells represents a fascinating approach to cancer treatment. Tumor cell specific antigens can be recognized by host immune system as short peptides bind with MHC (major histocompatibility complex) molecules [161]. As an exclusive tumor marker, survivin evidently activates T-cell immune response. Several survivin epitopes have been identified, which can induce cytotoxic T-lymphocyte (CTL) activity against cancer cells [162] (Fig. 3). In an interesting experiment, dendritic cells were infected with adenovirus-expressing survivin in the hope that endogenous overexpression of survivin may display some immunogenic short survivin peptides as human leukocyte class I or II antigens (HLA-I or II), and for avoiding any pro-oncogenic side effects, survivin dominant negative mutant was used [163]. Three HLA-A2 matching peptides of survivin have been identified against which T-cell immune response was induced by immunizing with these dendritic cells, and this CTL activity was found to be against cancer cells overexpressing survivin, such as MCF-7 breast cancer cell line [163]. Another study showed that CTL activity can be induced against B-cells transfected with survivin-expressing vector, which produced survivin-epitopes on the cell surface [164].

HLA-I restricted T-cell epitopes of survivin were also found in cancer patients that induced CTL response [165, 166]. Survivin-induced CTL have been tested against different malignancies, which are found to kill different HLA-matched tumors [167, 168]. A survivin-2B-derived HLA-24-restricted immunogenic peptide (aa-80-88: AYACNTSTL) has been identified that is recognized by CD8^+^ CTL [102]. On the basis of this peptide, a vaccine was developed and tested in a phase-I clinical trial on advanced stage patients with lung, breast and colorectal cancer, who were found to overexpress survivin-2B splice variant [169]. Another phase I trial of survivin-2B80-88 peptide vaccination has been started on HLA-A24-positive patients with advanced or recurrent OSCC, in which vaccine is being administered subcutaneously or intratumorally. Initial results have demonstrated the safety and marginal clinical effectiveness of this vaccine alone (UMIN000000976) [170]. However, subsequent clinical trials of survivin-based vaccination in combination with other drugs could be a promising therapeutic strategy to tackle advanced cancers (Table 2).

Survivin-specific antibodies have been found in the blood samples of cancer patients [164] and also reported in the tumors, but are absent in healthy specimens. This can be used for inducing humoral immune response. Purification of antibodies gives an opportunity to rationally design survivin-epitopes and consequently to develop cancer vaccines. A specific CTL response can be induced by presenting processed survivin-epitopes on dendritic cells [171]. Another approach to detect a specific T-cell response in cancer patients is based on the identification of tumor-specific survivin epitopes using ELISPOT assay [172, 173]. The presence of these survivin-epitope specific T-cells in tumor lesions indicated its tumor specificity [172]. Dr. Hooijberg and colleagues are exploring the possibility of using vaccination for the treatment of HNSCC patients based on dendritic cells targeting survivin [174]. His team was able to measure survivin-specific T cells ex vivo in peripheral blood and draining lymph node derived from HNSCC patients by using tetramer and ELISPOT analysis. Recently, a vaccine named SurVaxM is a peptidomimetic of survivin, which has entered in clinical trials (NCT01250470) (Table 2). These findings suggest that immunotherapy based on survivin may provide a novel approach for cancer treatment.

### Small molecule inhibitors

Small molecules targeting cancer signaling pathways offer an attractive strategy for controlling tumor growth. A range of small molecules and peptides has been identified that control tumor cell proliferation by targeting survivin (Fig. 3). Increasing understanding of molecular mechanisms that regulate survivin expression and function is providing opportunities for designing new molecules, which selectively intercept survivin functions in cancer cells.

#### Histone deacetylase inhibitors (HDACi)

Histone acetylation/deacetylation plays an important role in epigenetic regulation of transcription in eukaryotic cells [175]. Histone acetylation is tightly controlled by the balance of two groups of enzyme named histone acetyl transferases (HATs) and histone deacetylases (HDACs). HATs-mediated histone acetylation activates transcription through several transcriptional factors, whereas HDACs invert this reaction [175, 176]. This acetylation and deacetylation takes place on lysine residues at the N-terminal of core histones [176, 177]. Many cell cycle and apoptotic regulatory genes are regulated by the HATs/HDACs system. It has been suggested that deacetylation of survivin may potentially promote its cytoplasmic localization and allowing it to interact with α-tubulin. The α-tubulin binding site of survivin is located within the domain containing amino acids 99–142, including the lysine-129 residue, an target for deacetylation that play important role in survivin cytoplasmic localization, dimerization and stability [178, 179].

HDAC proteins have become pervasive cancer treatment targets due to their involvement in multiple signaling pathways that provide a survival advantage for tumor cells [180]. Many small molecules that block HDAC have been identified [181]. On the basis of structure, HDACi are classified in different groups, which include short-chain fatty acids (eg. VPA-valproic acid, NaB-sodium butyrate); cyclic tetrapeptides (eg. depsipeptide); hydroxamic acids (SAHA-suberoylanilide hydroxamic acid, TSA-trichostatin, Vorinostat, LAQ824, LBH529 and PXD101); and amides (MS-275, MGCD0103, CI-994). Clamydocin is one of the well-known HDACi, which has been tested to suppress proliferation of cancer cells [182]. Like other HDACi, treatments of clamydocin induced hyperacetylation of H3 and H4 histones in cancer cells and consequently arrest the cell cycle by activating expression of many important cell cycle regulatory genes, such as p21. It has been shown that clamydocin induces apoptosis by activating caspase-3. Fei et al. (2004) has demonstrated that clamydocin induces cancer cell apoptosis by proteasome-mediated degradation of survivin [183]. LAQ824 also induces apoptosis in cancer cells by down-regulating survivin protein [183].

It is known that TGFβ signaling decreases survivin expression in cancer cells in response to stress [184]. A report has suggested that HDACi belinostat represses survivin expression in TGFβ-dependent manner leading to cancer cell death. The early repression of survivin is mediated by proteasomal degradation, whereas the late suppression involves transcriptional repression of survivin expression [185]. Since survivin is expressed in cell cycle dependent manner, it is possible that cell cycle arrest may suppress transcription of the survivin promoter. Indeed, a report published showing that belinostat treatment increases level of p21^WAF1/CIP1^, which in turn activates survivin degradation mediated by suberoylanilide hydroxamic acid (SAHA)-dependent up-regulation of TGFβ [186]. A recent study shows that selective inhibition of HDAC2 by SAHA induces survivin downregulation in p53-dependent manner through MDM2 proteasomal degradation [187]. In prostate cancer cells, TSA treatment induced apoptosis, which is mediated by Cyclin B1/Cdc2-dependent degradation of survivin protein [188]. A clinical trial was conducted using SAHA in combination with fluorouracil, irinotecan hydrochloride and leucovorin calcium with the purpose to evaluate the safety and efficacy of SAHA along with these drugs in phase I and phase II, and to study alterations in TGF-β signaling and survivin expression (Table 2).

NF-kB contributes significantly to tumorigenesis by activating anti-apoptotic signaling pathways [189], leading to the up-regulation of anti-apoptotic proteins such as survivin. HDACi have been shown to suppress NF-kB signaling [190]. Kramer and colleagues suggested that induction of cancer cell apoptosis with the treatment of HDACi such as VPA is driven by hyperacetylation of Stat1 that allow its interaction with NF-kB and reduces NF-kB signaling [191], thus suppressing expression of NF-kB target genes including Bcl-XL, survivin, and Stat5. A study shows that valpromide (VPM), an amide analog of VPA that does not inhibit HDAC also potentiates cell death in cancer cells associated with decreased level of survivin indicating an alternative mechanism of VPA-mediated apoptosis [192]. Farnesylthiosalicylic acid, a Ras inhibitor was tested in combination with VPA. This treatment has shown a significant reduction in cancer cell proliferation due to the down-regulation of Ras and CPC proteins survivin and aurora B [193]. The characteristic features of CPC depletion such as cell cycle arrest, multinucleation and failure of cytokinesis were also reported. Interestingly VPA can also enhance anti-cancer effects of the other drugs such as cisplatin in in vivo studies on human tumor xenographt models [194]. An HDACi, OBP-801 (also known as YM753) and LY294002 (inhibitor of phosphatidylinositol 3-kinase, PI3K) act synergistically when used in combination to control growth of renal carcinoma cells. This combination treatment induces apoptosis mediated by survivin attenuation, which in turn activates caspase-3, -8 and -9 [195]. Recently, thailandepsin A (TDP-A) a novel class I HDACi has been tested for anticancer effects on thyroid carcinoma [196] and treatment shows inhibition of anti-apoptotic proteins survivin and bcl-2, which trigger caspase-dependent apoptosis.

#### Hsp90 inhibitors

Hsp90 is an important molecular chaperone for the accurate folding and stabilization of various proteins including survivin. It plays central role during cellular stress conditions. It has been discovered that interaction takes place between BIR domain of survivin and ATPase domain of Hsp90 [69]. Disruption of the survivin-Hsp90 complex by using targeted antibody or global inhibition of Hsp90 functions causes proteasomal degradation of survivin, consequently inducing mitotic defects and mitochondrial-dependent apoptosis. These results provide a direct link between cellular stress responses and survivin-mediated mitotic checkpoint. Thus, rational approaches to target survivin-Hsp90 complex, and consequent destabilization of survivin protein may be used in cancer therapeutics (Fig. 3).

Shepherdin is a first antagonist derived from survivin sequence Lys79–Leu87 to inhibit Hsp90-survivin complex formation. It is a cell-permeable peptidomimetic, which attenuates formation of Hsp90-survivin complex by competing with survivin to bind at the same site on Hsp90 [197]. Interestingly, despite being derived from survivin sequence, shepherdin is also destabilized by many other Hsp90-binding proteins, such as Akt, telomerase and CDK6 in favor of inducing apoptosis. As reported in many studies, shepherdin can trigger both caspase-dependent and caspase-independent cell death pathways. For example, shepherdin treatment suppressed growth of prostate and breast tumors in xenograft models, and showed minimal normal cell toxicity [197, 198]. For overcoming limitation of peptide therapy, a recombinant adeno-associated virus (rAAV) was developed to deliver shepherdin in cancer cells. rAAV transduction significantly decreased the level of survivin and induced caspase-dependent apoptosis in NSCLC cells [199]. Blocking of survivin-Hsp90 complex formation also sensitized resistance cells to conventional drugs. For instance, treatment of resistant chronic myelogenous leukemia cells with shepherdin enhanced cell death induced by hydroxyurea and doxorubicin [200]. Shepherdin is currently in the pre-clinical phase [201]. Furthermore, a number of other Hsp90 inhibitors such as 17-DMAG, 17-AAG, Isoxazolo(aza)naphthoquinones, NVP-AUY922 and NVP-BEP800 are currently undergoing discovery and clinical trial phases [202–205].

#### Cyclin-Dependent Kinase Inhibitors (CDKi)

Phosphorylation of survivin on Thr34 during mitosis is a key to its functional activation [206]. Tumor cells could acquire resistance against paclitaxel due to the induction of survivin phosphorylation at the time of cell cycle arrest. Therefore, CDKi were tested in cancer cells arrested in mitosis with paclitaxel to inhibit survivin phosphorylation, which paclitaxel-induced apoptosis [206, 207]. Flavopiridol (CDKi) and purvalanol A (p34cdc2 inhibitor) are tested in this in vivo study (Fig. 3), in which cells escaped from paclitaxel-mediated cell cycle arrest due to loss of survivin functions [206]. A novel CDKi, NU6140 (4-(6-cyclohexylmethoxy- 9Hpurin-2-ylamino)-N,N-diethyl-benzamide) has been tested on Hela cancer cells and results suggesting that inhibition of survivin phosphorylation is a potential underlying mechanism by which CDKi induced taxol-mediated apoptosis [208]. A pharmacological study was conducted to evaluate the effects of a new CDKi roscovitine (ROSC) on doxorubicin resistant human multiple myeloma cells [209], in which exposure of ROSC induced hypoploidy condition, suggesting cells were undergoing apoptosis. Destabilization of survivin was involved in this ROSC-induced apoptosis. Ibulocydine (an isobutyrate prodrug of the CDKi, BMK-Y101) have shown strong anti-proliferative effects in hepatocellular carcinoma (HCC) xenograft mouse [210], which is again mediated by the down-regulation of survivin and other anti-apoptotic proteins.

#### Other inhibitors

Several other molecules have been developed to target survivin for clinical applications. Terameprocol (meso-tetra-O-methyl nordihydroguaiaretic acid) drug has shown to inhibit transcription of specific protein 1 (Sp1) regulated genes, such as survivin and cdc2 [211], which subsequently activates mitochondrial-apoptotic pathway. Systemic treatment of this drug suppressed tumor growth in human xenograft mouse model [212]. However, Sp1 can regulate transcription of several other genes, and it is possible that terameprocol suppresses tumor growth by not only survivin-mediated pathway, but also involving other pathways. A phase-I and II clinical trials of terameprocol are being conducted on patients with refractory solid tumors (NCT00664586), lymphoma and leukemia (NCT00664677) (Table 2). Since interaction of survivin with Smac/DIABLO plays a crucial role in anti-apoptotic functions of survivin, an analog, 5-deazaflavin was designed to block this interaction. Treatment with this analog induces apoptosis in cancer cells by activation of stress pathways [213].

YM155 is an imidazolium-based small molecule, which selectively inhibits survivin in many different cancer cell lines, such as OSCC, ESCC, HNSCC, NSCLC, colon and cervical carcinoma [214–217]. Studies show that YM155 function as a transcriptional suppressor for survivin promoter [67]. Glaros and colleagues proposed that YM155 could precede selective transcriptional inhibition of survivin due to DNA damage induction [218]. The YM155 discoverer research group at Astellas Inc. found that YM155 directly interacts with a transcription factor ILF3, which plays an important role in survivin transcription in association with p54^nrb^ [219]. Specifically YM155 binds with C-terminal region of ILF3, which is also critical for survivin expression [220]. Recently, Sachita et al. [221] demonstrated that YM155 causes apoptosis of human oral cancer cell lines Sp1-mediated downregulation of survivin. In vitro studies showed strong anti-proliferative activity of this molecule and a nanomolar concentration range is enough to clinically achievably dose. YM155 have also shown to induce non-apoptotic cancer cell death via poly-ADP polymer (PARP-1) activation and AIF translocation from the cytosol to the nucleus [216]. Genetic knockdown of PARP-1 or AIF abrogated YM155-induced cell death in esophageal cancer [216]. In HNSCC, YM155 treatment triggered both mitochondrial and receptor-mediated apoptosis. YM155 significantly induced autophagy in HNSCC cells by upregulating Beclin1 that leads to cell death [217]. Further, many in vivo studies showed a strong suppression of tumor growth in a variety of tumor xenographt models, such as NSCLC, lymphoma, prostate cancer, HNSCC, ESCC [216, 217, 222]. Several clinical trials of YM155 are ongoing with different cancer patients (Table 2). Early results have shown safety and efficacy of YM155 in phase I [223] and phase II [224] clinical trials on NSCLC patients. In another phase I clinical trials on patients with different cancers, YM155 showed a good response in preliminary results [225]. A phase II clinical trial of YM155 is ongoing on patients with advanced melanoma and B-cell lymphoma [226]. Also, a phase I clinical trial of a combination therapy using YM155 and docetaxel is in progress for patients with prostate cancer (NCT00514267). These studies are indicating that not only molecular therapy but small compounds and peptides targeting survivin provide potent antitumor effects.

## Conclusions

Survivin is one of the top five tumor markers, exclusively overexpressed in most cancers, making it an ideal target for cancer therapeutics. High level of survivin help in the promotion of cancer development through contributing via a wide range of cellular mechanisms including growth and apoptotic pathways. In cancer patients, an elevated level of survivin is often associated with poor prognosis and therapy resistance, and it also promotes metastasis in cancer cells. Several gene silencing studies have clearly demonstrated a crucial role of survivin in cancer development. Currently, most of the cancer treatment protocols are an involved combination of surgery, chemotherapy and radiotherapy, but even after substantial growth in this direction, patient survival rates have not changed much. Therefore, lots of studies are being conducted to explore the possibility of using molecular targeting therapies along with conventional therapies to tackle the menace of cancer. An increasing number of clinical trials are taking place with the selected patients based on validated biomarker-enrichment (Table 2). Survivin is considered an excellent molecular target for cancer treatment, and several therapeutic strategies, such as gene silencing, immunotherapy, and small molecule inhibition have been designed and tested in different pre-clinical and clinical studies. In the future, administration of survivin-targeted agents alone or in combination with conventional therapies may generate a novel therapeutic strategy against cancer.
